# Residential factors associated with mental health in United States Veterans, Air Force military, and Air Force employees

**DOI:** 10.1016/j.indenv.2025.100103

**Published:** 2025-06

**Authors:** Andrew J. Hoisington, Christopher A. Stamper, Molly E. Penzenik, Meredith Reitter, Elizabeth J. Kovacs, Nazanin H. Bahraini, Lisa A. Brenner

**Affiliations:** a Department of Physical Medicine & Rehabilitation, University of Colorado Anschutz Medical, Campus, Aurora, CO, USA; b Rocky Mountain Mental Illness Research Education and Clinical Center (MIRECC), Rocky, Mountain Regional VA Medical Center (RMRVAMC), Aurora, CO, USA; c Department of Systems Engineering & Management, Air Force Institute of Technology, Wright-Patterson AFB, OH, USA; d Department of Surgery, Division of GI, Trauma, and Endocrine Surgery, University of Colorado Anschutz Medical Campus, Aurora, CO, USA; e Alcohol Research Program, University of Colorado Anschutz Medical Campus, Aurora, CO, USA; f Department of Psychiatry, University of Colorado Anschutz Medical Campus, Aurora, CO, USA; g Veterans Health Administration, Eastern Colorado Health Care System, Rocky Mountain Regional Veterans Affairs Medical Center (RMRVAMC), Aurora, CO, USA; h Center for Neuroscience, University of Colorado Anschutz Medical Campus, Aurora, CO, USA

**Keywords:** Residences, Mental Health, Survey, Military, Indoor Environment, Building Quality

## Abstract

Individuals in Westernized countries spend most of their time indoors. However, exploration of residential building factors that may influence occupants’ mental health is limited in scientific literature. The purpose of this study was to explore investigator’s perceived areas of importance in residences to mental health via survey methods. To that end, we administered the Housing, Occupancy, Materials, and Environment (HOME) survey to assess factors that may influence mental health to those working in the United States (US) Air Force (n = 230) or past military members, US Veterans (n = 180). Self-reported mental health surveys were also administered to the Air Force (RAND 36-Item Short-Form) and Veterans (36-Item Short-Form survey version 2, Patient Health Questionnaire-9). The residential question that correlated to the most mental health measures for both groups was an ability to adjust indoor climate, with positive correlations. Other correlations between residential questions and health scores across the two groups were dissimilar, indicating the residential factors of importance to mental health may be variable across an individual’s life. For example, multiple positive correlations between mental health measures and nature in the older Veteran group support robust and support previous results on the importance of nature to older adults. Overall, this study provides a basis for future research and targeted clinical interventions that can quantify and positively impact the home environment and improve mental health outcomes.

## 1. Introduction

It is well established that individuals spend a majority of their time indoors. [1] More specifically, in the United States (US) the average time individuals spend at their residence is 73 % of weekdays and 82 % of weekends. [2] Nevertheless, additional knowledge is still needed in the area of the built environment and mental health, a challenge due to variability in residential designs, conditions, and neighborhood characteristics. [3] Additionally, the low population density of 2–3 individuals per house in the US presents research design-related challenges deploying potentially expensive measurement devices. As such, research studies are often conducted at the neighborhood or census tract levels, using readily obtainable survey information and data from central measurement devices, which may overlook important details at a residence or individual level.

In terms of building surveys (i.e., neighborhood, census) the US Environmental Protection Agency (EPA) developed the Building Assessment Survey and Evaluation (BASE) study in the 1990s. This study included standardized mechanisms for sample collection, collection of building characteristics, and a 10-page questionnaire evaluating occupants’ perceived indoor air quality and health symptoms. [4] Several years after the BASE study, the Center for the Built Environment (CBE) at the University of California Berkley, released a fee-based occupant survey that has been used extensively in 897 buildings with 93,662 participants across 11 countries. [5] Both the BASE and CBE surveys were developed for workplace environments which may not translate well to residential settings. In 2002, the World Health Organization launched the Large Analysis and Review of European Housing and Health Status (LARS) study, which included a self-administered survey on housing and neighborhood conditions and an occupant health questionnaire, involving over 8000 residents across 8 European cities. [6–8] Surveys are readily available in the built environment. For example, surveys in the built environment have been developed to investigate topics such as occupant satisfaction, [9–14] post-occupancy evaluations, [15–18] noise, [19–22] indoor air quality, [23–25] and building design choices. [11] A survey that specifically focuses on mental health and the indoor environment is a less studied area of research.

The indoor environment can influence the mental health of occupants in multiple ways. In a residential environment, high concentrations of mold spores, [26,27] particulates, [28–30] and other air pollutants [31–34] could cause a biological response that may exacerbate existing or lead to new onset of psychiatric conditions. [35–37] Additionally, light plays a crucial role in mental health; [38,39] reduced natural light during certain seasons can trigger chemical changes in the brain that can contribute to Seasonal Affective Disorder, [40,41] with artificial light therapy often providing relief from symptoms. [42,43] More broadly, the lack of windows and natural light [44–46] as well as limited views of nature [8,47] can result in negative psychological outcomes. Other residential building factors including ventilation, [48–50] housing quality, [51,52] and housing density [53,54] also contribute to overall occupant satisfaction levels.

Surveys offer a low cost and practical methods for obtaining person-level information. Obtaining survey data has been aided through online programs such as Google Forms, Qualtrics, Research Electronic Data Capture (REDCap) program, and SurveyMonkey, which simplify the design and distribution of questionnaires. [55,56] Such methods were used to collect data for this study with the aim of investigating aspects of the residential environment that are associated with occupant mental health. To conduct this study, we developed the Housing, Occupancy, Materials, and Environment (HOME) survey, a 21-item self-report questionnaire that can be administered online to augment study designs and other surveys of residential settings. This is a new survey that includes items related to the residential environment with a specific focus on relating indoor factors to mental health outcomes. The aims of this study were to: (1) identify factors in the residential environment that correlate to perceived mental health; (2) compare associations between residential and mental health factors among US military workers and Veterans of different ages; and (3) explore the feasibility of using this survey for the first time.

## 2. Methods

### 2.1. Participants

Two hundred and thirty participants in the present study were active-duty US Air Force (AF) military members or AF civilian personnel. Recruitment for this survey was conducted by AF leadership with an email invitation drafted by the study team. The email addresses and any identifying information was not known to the study team. The participants were asked to complete an anonymous Google Forms survey online from September to December 2019. In addition to the HOME survey, participants provided responses for demographics and the RAND 36-Item Short-Form survey (RAND SF-36). [57] Participation from these individuals was approved by the Air Force Institute of Technology Institutional Review Board (IRB #REN2019024R).

One hundred and eighty participants in the study were Veterans in the United States-Veteran Microbiome Project (US-VMP) study occurring at the Rocky Mountain Mental Illness Research, Education, and Clinical Center (MIRECC) for Suicide Prevention. [58] Briefly, the study is a comprehensive analysis of Veterans to include clinical surveys, psychometrically sound psychological measures, self-reported measures, demographics, biological samples, and access to participants’ electronic medical records. Enrollment for US-VMP started in May 2016 and collection of the survey data presented in the present study was from March 2018 to February 2020. Survey response was through the REDCap program to include a demographics questionnaire, 36-Item Short-Form survey version 2 (SF-36v2) [59], and the Patient Health Questionnaire-9 (PHQ-9) [60]. The US-VMP study was approved by the Colorado Multiple Institutional Review Board (COMIRB #15–1885).

### 2.2. Measures

The RAND SF-36 measures perceived health through two summary measures and eight health scales to include physical functioning, role limitations due to personal problems, role limitations due to emotional problems, energy/fatigue, emotional well-being, social functioning, pain, and general health. Composite physical and mental component scores were determined based on previously reported methods. [61] The SF-36v2 provides a brief, yet comprehensive measure of perceived health with eight scales yielding two summary measures: physical and mental component scores. The eight scales measure physical functioning, role-physical, bodily pain, general health, vitality, social functioning, role-emotional, and mental health. The SF-36v2 is similar to the RAND SF-36 with changes in question wording, responses, and scale names. Proprietary software from QualityMetric was used to calculate the SF-36v2 summary and scale measures. Higher scores in the RAND SF-36 or the SF-36v2 indicate healthier self-reported conditions. The PHQ-9 is a self-reported measure of depressive-related symptoms with higher scored potentially indicative of increased levels of depression.

The HOME survey is predominantly comprised of dichotomous questions supplemented by multiple choice, Likert scale, or open-ended questions (see Supplemental File for full survey). The survey was developed after a thorough literature review to ascertain how factors in residences could theoretically correlate to mental health outcomes (See Supplemental Table 4 for more detailed information). [3,35,62–64] Although the current study was not a validation study, we briefly evaluated the HOME survey after combining the AF and Veteran respondents. First, correlations were measured between four questions selected *a priori* that theoretically may be related, measured through Pearson’s Chi-squared test or paired *t*-test in R (Table 1). Second, readability and grade level determination for the HOME survey was performed online. [65] Only the full questions were used for these measures since readability scores are determined through computational algorithms that include common words, syllables, word length, and sentence length. Finally, any questions that were missing responses by over 5 % of participants were noted as potentially problematic or confusing.

### 2.3. Statistical analysis

Statistical analysis was conducted in the open software R v. 4.2.2 [66] with a two-tailed alpha level of 0.05 and multiple comparisons were corrected using false discovery rate (FDR). [67] Prior to development of correlation matrixes, scoring for seven questions in the HOME survey were inversed or reversed to match theoretical expectations in relation to mental health. Specifically, questions with adjusted scale included: people living with participant was inversed (Q2); smoking indoors was reversed (Q6); burning incense was reversed (Q7); living within one mile of highway was reversed (Q12); basement in home was reversed (Q15), water damage was reversed (Q16); renting or owning was reversed (Q18); natural lighting was reversed (Q20); and adjusting indoor climate was reversed (Q21). The directionality of the questions was confirmed through the Cronbach alpha in R package “psych”. Correlation matrices were developed through the R package “sjPlot” with default settings and Pearson correlation coefficient. Confirmatory factor analysis was completed using the R package “lavaan” based on three constructs to include: (1) Indoor Air Quality from questions 6,7,10,12, 16; (2) Nature from questions 8, 11–13, 17, 20; and (3) Home Quality from questions 2, 9, 12, 14, 16, 17, 18, 20, and 21. Additionally, principal component analysis (PCA) for all questions was conducted using the R package “pcaMethods” with default settings. Questions that grouped together in PCA were tested for homogeneity with the Kuder-Richardson formula.

## 3. Results

### 3.1. Demographics characteristics and survey summary

Demographic information collected for all 410 participants and the subset of Air Force and Veteran groups are shown in Supplemental Table 1. Our study population differed from the US population in several factors, including sex (male 76.8 % this study vs. 49 % in US population [68]) and education level (high school graduate or higher 99.8 % this study vs. 88.5 % in US adult population [69]). These differences are reflective of US military historical demographics on sex and a requirement of a high school degree to join the military. The residential factors from the HOME survey are shown in Table 2. Respondents in the present study moved more frequently per year than other individuals in the United States (approximately 43 % this study vs. 13 % in US [70]), had higher level of renting (46.3 % this study vs. 34.8 % in US [70]), and had less occupants in the home (mean 2.4 this study vs. 3.1 in US [70]).

A total of 230 participants from the AF took the RAND SF-36 survey and scored in eight health components (mean ± standard deviation) to include: physical functioning (88.5 ± 19.6), role limitations due to personal problems (83.3 ± 31.0), role limitations due to emotional problems (82.4 ± 33.0), energy/fatigue (55.3 ± 19.8), emotional well-being (73.8 ± 16.9), social functioning (75.5 ± 18.5), pain (75.0 ± 21.3), and general health (70.5 ± 18.1). The sub score for mental component score (MCS) was 48.4 ± 9.9 and the physical component score (PCS) was 52.1 ± 8.5. A higher score indicates better health status for each component. One hundred and eighty Veteran participants completed the SF-36v2 survey that was scored in eight health components (mean ± standard deviation) to include: physical functioning (63.9 ± 26.9), role physical (56.6 ± 30.3), role emotional (61.8 ± 29.2), vitality (44.0 ± 23.2), social functioning (56.7 ± 30.8), bodily pain (47.6 ± 25.1), mental health (58.7 ± 22.8), and general health (53.4 ± 24.0). The two sub scores were 43.5 ± 10.4 for PCS and 41.4 ± 13.6 for MCS. Veterans were also given the PHQ-9, a measure of depression, and scored 9.3 ± 6.8.

### 3.2. Air force military and civilians associations between residential factors and mental health

The eight components and two subcomponent scores from the mental health survey were compared to the HOME survey questions. The ability to adjust indoor climate was positively correlated with mental health scores (Fig. 1). Air Force participants who could open a window at home scored significantly higher in PCS, general health, physical functioning, social functioning, and pain (Fig. 2). Participants reporting adequate privacy in their homes scored significantly higher in five component scores: MCS, general health, social functioning, emotion well-being, and pain (Fig. 3). Only two other questions were significantly associated with component scores, natural light and energy/fatigue category (*p_fdr_* = 0.022, higher natural light had lower energy/fatigue scores) and history of water damage and general health (*p_fdr_* = 0.035, reported water damage positively associated with lower general health scores). HOME survey questions without correlations to any of the RAND SF-36 component scores included people living in home (Q2), burning incense or candles (Q6), 50 % or more windows looking upon natural setting (Q7), living within one mile of highway (Q12), living within 0.5 miles of open greenspace (Q13), basement (Q15), and type of home (Q19). Supplemental Table 2 contains correlations between all mental health measures and HOME responses.

### 3.3. US Veterans associations between residential factors and mental health

In correlation analysis, the HOME question that was observed to be significant for the most number of health survey measures was the question that asked about the Veterans ability to adjust their indoor climate. The direction of correlations were positive, indicating participants that reported more ability to adjust indoor climate had improved mental health (Fig. 4). The next two questions that correlated with more mental health scores were pictures of nature and proximity to parks (Figs. 5 and 6). The only other question that had significant correlation was a history of water damage to general health (*p_fdr_* = 0.032, reported water damage positively associated with worse general health scores) and PHQ-9 score (*p_fdr_* = 0.040, reported water damage positively associated with depression scores). HOME survey questions without correlations to any of the SF-36v2 component scores included smoking in home (Q6), burning incense or candles (Q7), 50 % or more windows looking upon natural setting (Q11), living within one mile of highway (Q12), adequate privacy (Q14), renting or owning a home (Q18), type of home (Q19), and natural lighting (Q20). See Supplemental Table 3 for all correlations between all mental health component and subcomponent scores and HOME responses.

### 3.4. Comparison between AF and Veteran groups

The AF and Veteran groups provide different perspectives for the assessing the HOME survey results. The AF cohort inclined to be younger (49 or younger 76.1 % AF vs. 53.3 % Veterans), married (72.2 % AF vs. 48.3 % Veterans), employed (full-time employment 99.6 % AF vs. 26.6 % Veterans), and without a homeless experience (lifetime homeless 3.5 % AF vs. 24.8 % Veterans). Military service requires frequent relocations, therefore a mean of 4.4 residences in the last 10 years for the AF group was unsurprising. Veterans relocating to new residences at nearly the same rate (mean 4.2) was unexpected. Air Force respondents’ residences, as compared to the Veteran respondents, were constructed more recently (1997 AF vs 1982 Veterans), on smaller lots (0.25 acres or less 51.6 % AF vs. 37.5 % Veterans), had fewer basements (10.9 % AF vs. 52.2 % Veterans), and had less smoking indoors (5.7 % AF vs. 28.3 % Veterans). In terms of mental health, the Veteran group scored lower for all component scores, as compared to the AF group. However, non-parametric ranking of the mental health subscores were the same for the two groups to include the same: (1) top three component scores of physical function, personal role limitation, emotional role limitation; (2) lowest component score of vitality and energy/fatigue; and (3) physical component score was higher than mental component score. The ability to adjust indoor climate was the most correlated factor for both the AF and Veteran groups. Other correlations were different between the groups. The AF group’s mental health scores had the next strongest correlation with the ability to open windows and adequate privacy, while the Veteran group mental health scores were more correlated with having pictures of nature and proximity to a park.

### 3.5. HOME survey assessment

The survey had a Flesch reading ease score of 81.7, indicating the survey was easy to read. The Gunning Fog Index, an estimate of grade level proficiency, was 7.9. The SMOG index, another measure of years of education to understand a text, was 6th grade. Since this was the first use of the HOME survey, we analyzed several factors post-survey to evaluate the resulting data. Significant correlations were observed for those questions selected *a priori* to include: owning a home (Q18) and conducting major repairs (Q9) (Chi-Squared, *p* < 0.001); history of water damage (Q16) and a basement (Q15) (Chi-Squared, p = 0.011); and smoking indoors (Q6) and burning incense (Q7) (Chi-Squared, p = 0.011). Additionally, the size of the residence (Q3) was also significantly correlated to the type of home (Q19). Specifically, a single-family detached home (mean 2176 square foot) was significantly different from apartments (mean 872 square feet; pairwise *t*-test *p_fdr_* <0.001), and multiple dwelling units (mean 1590 square foot; pairwise *t*-test *p_fdr_* < 0.001).

Two questions in the HOME survey were unanswered in over 5 % of the surveys, indicating a difficult or confusing question. The highest percentage of blank responses was on the question of house age (Q6), with a 20.2 % blank rate. In our study, 53.7 % of respondents owned their home, lower than the US average of 64.8 % homeownership. [70] The age of the home was left blank in 39 % of renters’ responses and 3.2 % of owners’ responses. Future surveys might reduce this problem by providing a multiple-choice response for the time period the residential unit was built (see HOME V2 in Supplemental File). The other unanswered question was the square footage of the home (Q3) with 5.9 % of surveys unresponsive for that question. The percentage of respondents with blank on square footage of the home was similar in renters and owners, indicating an overall difficulty in estimating residential square feet. Again, a range might be more appropriate in future surveys.

Analysis of both cohort results from the HOME survey revealed a multi-dimensional construct (Supplemental Figure 1) in which 23.9 % of the variance was explained in the two most prevalent dimensions. Those two dimensions were most related to renting or owning the home (Q18), adjusting climate (Q21), natural lighting (Q20), renovations (Q9), history of water damage (Q16), and type of home (Q19). From principal component analysis, we identified four potential groupings of residences based on survey response. However, measurements of internal consistency reliability from Kuder-Richardson (K-20) coefficient of equivalence ranged from 0.11 to 0.25, indicating the groupings were not reliable, supporting a multi-dimensional survey design that lacks subgroups.

## 4. Discussion

A survey developed by this research team was used to investigate the association between the residential environment and mental health. The short survey was feasible to implement and provided the research team with actionable information. With over 400 respondents in the AF and Veteran groups, we identified the most positive correlations between mental health scores and the ability to adjust indoor climate. The AF group had multiple significant health scores correlated to an ability to open windows and adequate privacy. In contrast, for the Veteran group, mental health scores were positively associated with having pictures of nature and proximity to greenspace.

Indoor temperature can influence health outcomes in the built environment (see review [71]), a topic studied primarily in public buildings [72–74] in which occupants are often unable to personalize the indoor climate. The inability to adequately control temperature could impact psychological health through a lack of self-efficacy [75] that contribute to increased levels of stress. [76] Though this study did not directly measure reasons for lower temperature control, our findings indicate that individuals who could adjust their indoor climate reported better mental health scores. A lack of control may be rooted in economic difficulties (i.e., inability to afford heating/cooling), apartment or dorm living conditions, or sharing a residence with occupants who prefer different temperatures. Indeed, our results suggest that higher control over the environment correlates with owning a residence and living in a detached single-family unit. Future studies in this field would benefit from monitoring the indoor temperature and comparing to participant ideal temperature.

The importance of nature, particularly valued by older adults, [77] could be tied to positive health outcomes [78] through increased levels of exercise, [79] engagement with others, [80] or access to natural light. [81] Psychologically, connections for improved mental health in relation to nature have been explored in the attention restoration theory [82] (i.e. nature restores ability to concentrate) or the biophilia hypothesis [83] (i.e., natural human reaction to other life is comforting and results in lower stress). Correlation between nature (e.g., proximity to greenspace, having pictures of nature) and improved scores in mental health were observed to be significant only in the Veteran group. This difference could be related to the increased age and lower employment levels in the Veteran group, theoretically resulting in increased time at a residence to enjoy factors related to nature.

Our findings suggest that residential environment and impact on mental health outcomes may shift over the life course and, therefore, factors of importance can change over time. Older adults have physical conditions (e.g. hearing, vision, communication, mobility, memory) that can cause challenges in social situations [84] and result in lower health scores. Additionally, older adults have increased time in their residence. [85] In support of that theory, a greater number of people living in the home was significantly correlated to multiple mental health measures in the Veterans but not in the AF results, despite on average a 0.5 higher mean occupancy in the AF. The family living situation was unassessed, yet we expect the AF group had more immediate family living in their residences with a majority of AF group married and under 40 years old. In addition, the AF group had no significant correlations with proximity to greenspace, a recognized important factor in multiple previous health and built environment studies. [86–88] The AF group, as well as other full-time employed and middle-aged groups, may have more ability to travel to greenspaces and as such, an immediate proximity is not as important for health, as compared to the Veteran group.

This study had some limitations. The use of two different health survey measures (SF-36v2 and RAND SF-36) limited direct comparison of results between the two groups. However, both the RAND SF-36 and the SF-36v2 are psychometrically-sound self-reported surveys that are available for use by researchers investigating the built environment and mental health. Additional self-reported validated psychological measures are available, many without cost and translated to multiple languages, depending upon the research question (see our previous review [3]). Our study participants were all associated with the military that might not generalize to other populations given the lower percentage of females, an increase frequency of moving, and higher levels of renting compared to home ownership. Additionally, the survey measures were all self-reported which could introduce bias. The study was conducted with a convenience sample and lacked a defined control group. Factors such as socioeconomic status, medical history, age, gender, and ethnicity, which were not accounted for, might also influence results. Finally, while correlations were observed, causation cannot be determined. As such, we cannot state that any of these residential factors cause new or worsening mental health related outcomes. Future trials (e. g., light, noise, air quality) are warranted to determine causative residential factors that influence mental health. In addition, further efforts to evaluate the psychometric properties of the HOME survey are warranted. Future studies would also benefit from indoor measurements (i. e. airborne constituents, light, noise, temperature, room occupancy [89]) and verification of psychological health by medical professionals.

## 5. Conclusion

Research on the indoor environment and mental health has been limited, despite one in five Americans being diagnosed with mental health disorders in the past year. Socially disadvantaged groups and those with higher rates of negative mental health conditions, such a US Veterans, are ideal candidates for initial residential based surveys and future interventions. This study showed that indoor environmental factors were associated with mental health outcomes and that some of these correlations differed between the AF and Veterans groups. Future studies can use the HOME survey to gather initial data, paving the way for clinical interventions that could potentially improve the well-being of many individuals.

## Supplementary Material

Supplemental File

## Figures and Tables

**Fig. 1.**
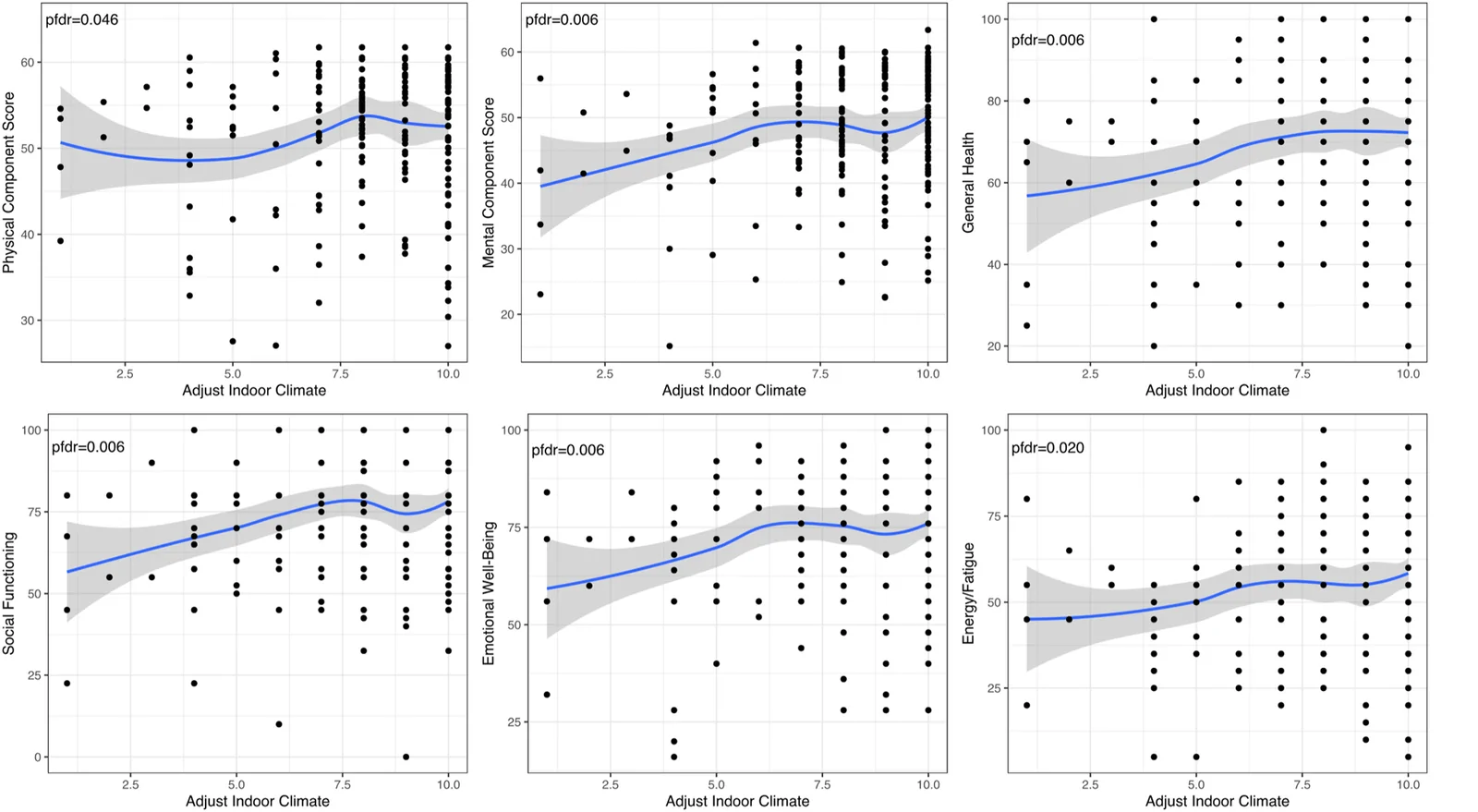
Air Force statistically significant RAND SF-36 component scores to question on ability to adjust indoor climate (Q21). *p*_*fdr*_ determined from Pearson correlation coefficient, adjusted for multiple testing between the component scores through false discovery rates. Displayed lines from local estimated scatterplot smoothing (LOESS) with confidence interval at 0.95.

**Fig. 2.**
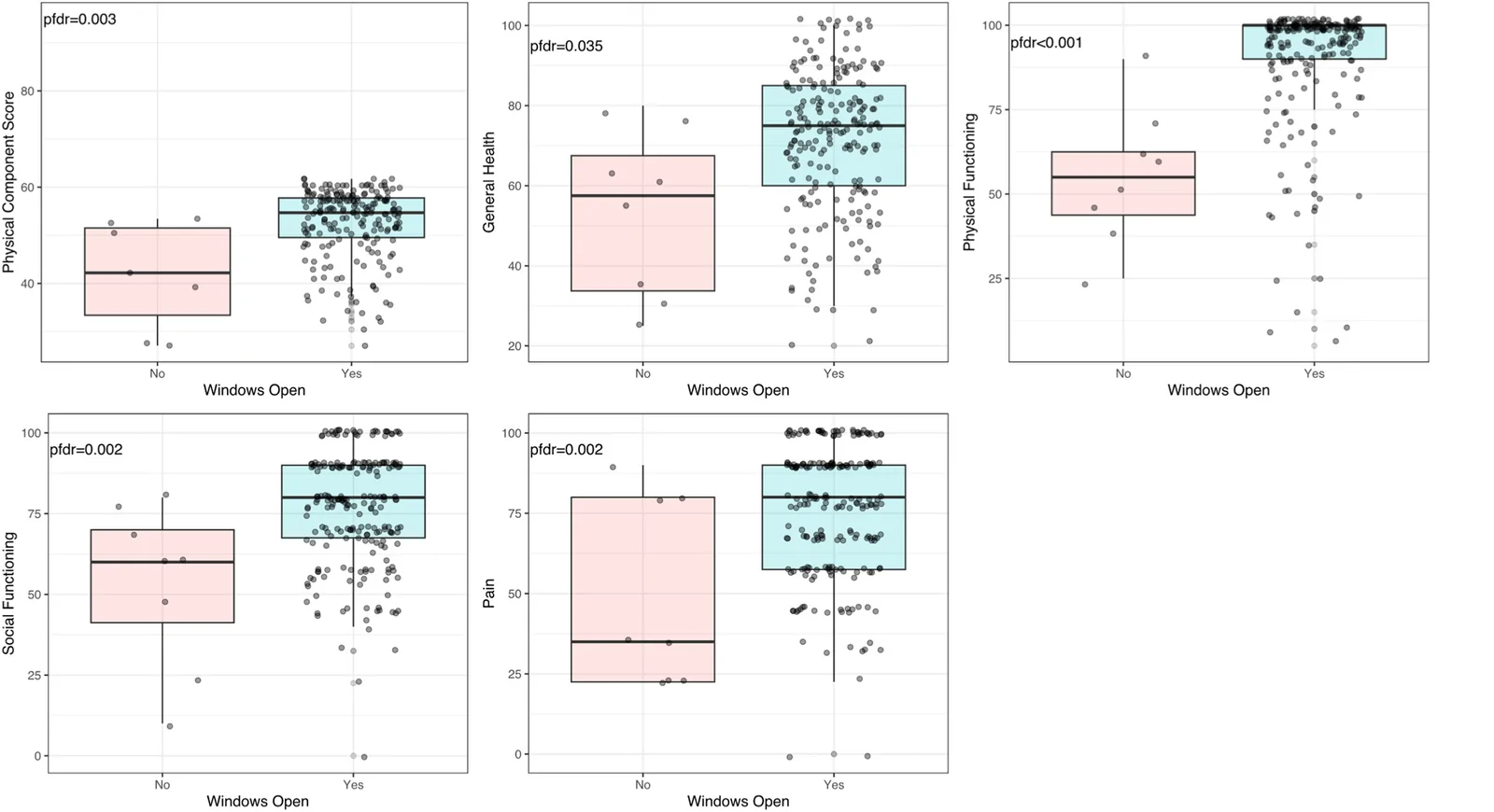
Air Force statistically significant RAND SF-36 component scores to question on ability to open windows (Q10). *p*_*fdr*_ determined from pairwise *t*-test, adjusted for multiple testing between the component scores and survey questions through false discovery rates.

**Fig. 3.**
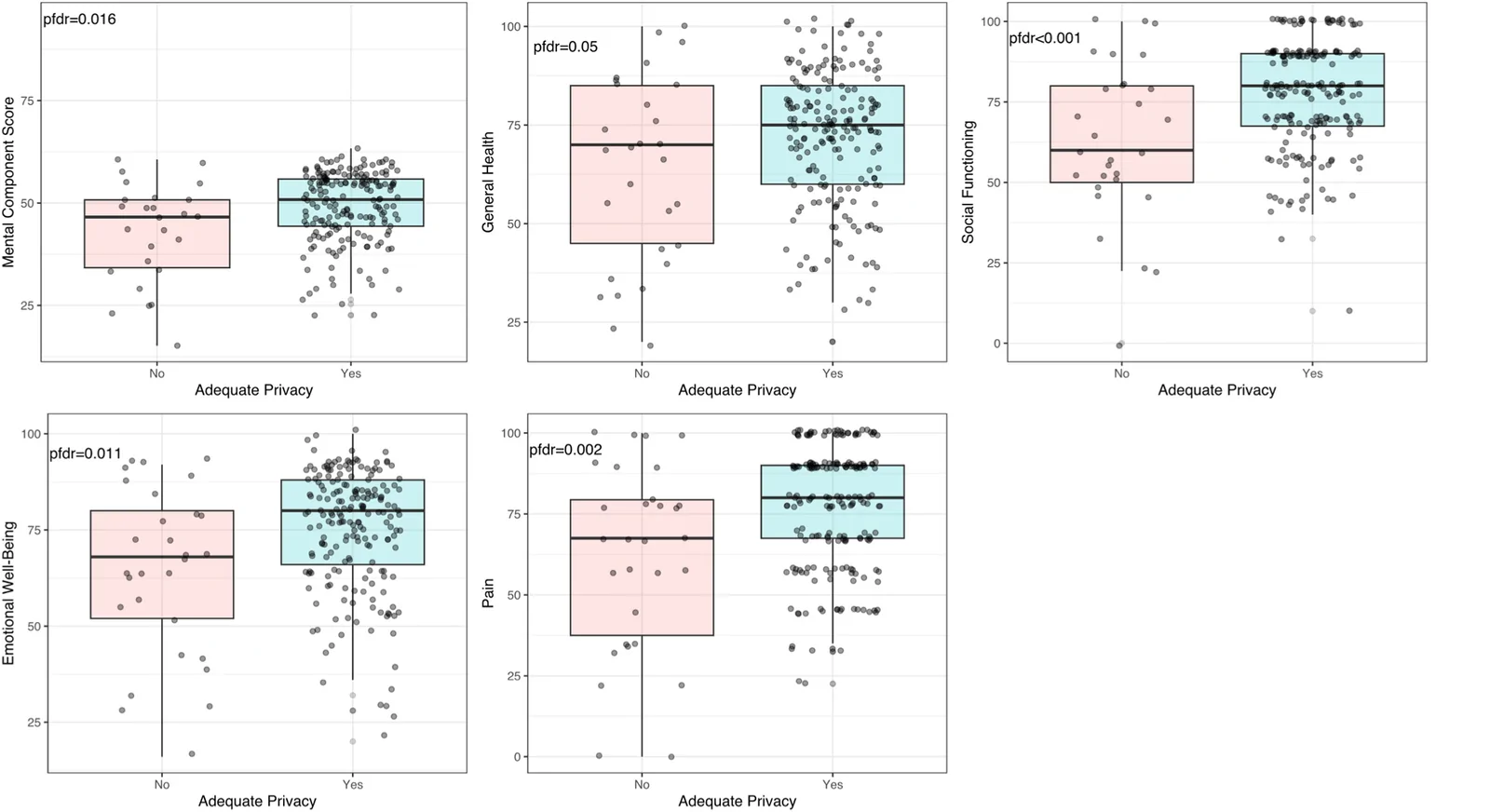
Air Force statistically significant RAND SF-36 component scores to question on adequate privacy (Q14). *p*_*fdr*_ determined from pairwise *t*-test adjusted for multiple testing between the component scores and survey questions through false discovery rates.

**Fig. 4.**
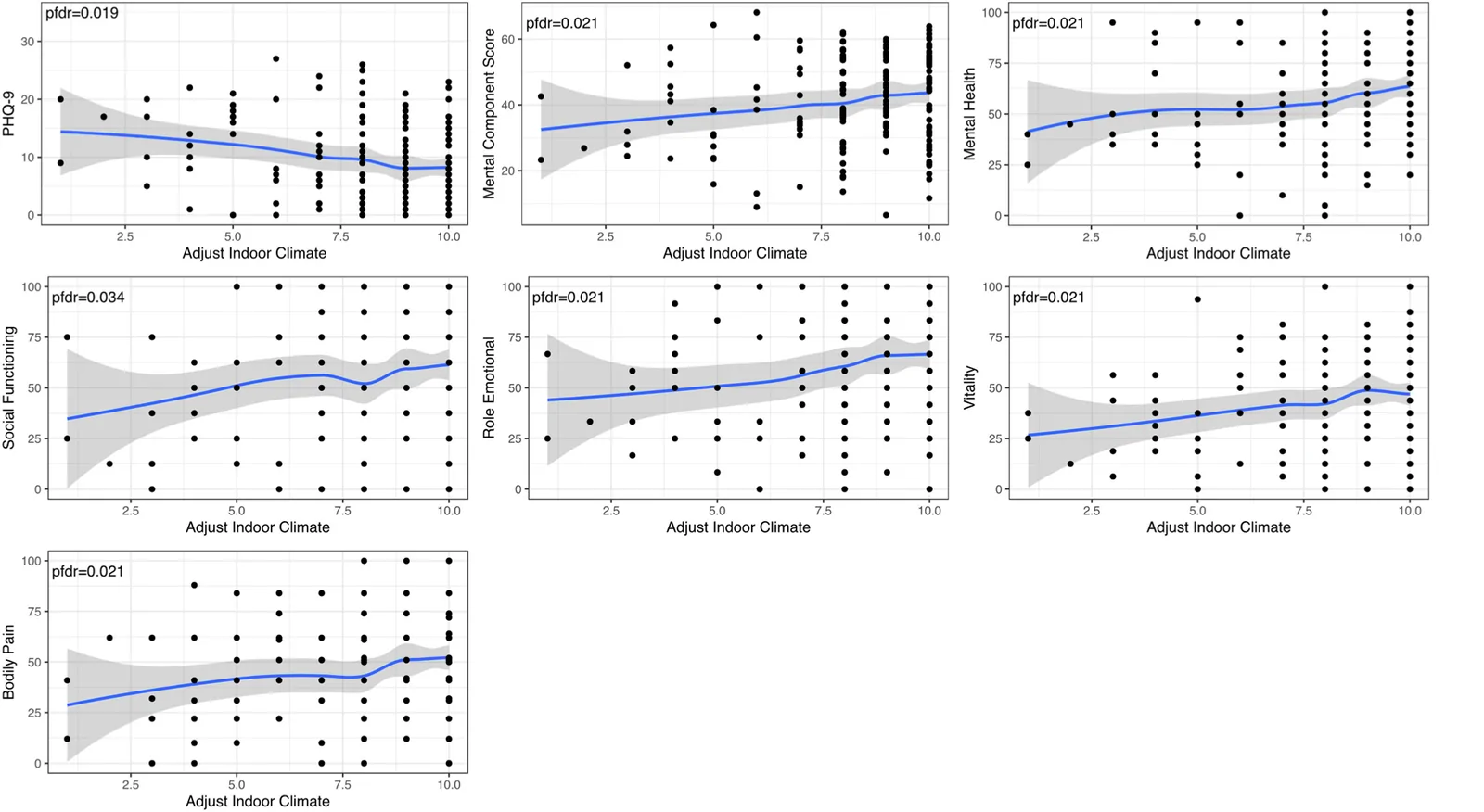
Veteran statistically significant SF-36v2 component scores and PHQ-9 total score to question on ability to adjust indoor climate (Q10). *p*_*fdr*_ determined from Pearson correlation coefficient adjusted for multiple testing between the component scores through false discovery rates. Displayed lines from local estimated scatterplot smoothing (LOESS) with confidence interval at 0.95.

**Fig. 5.**
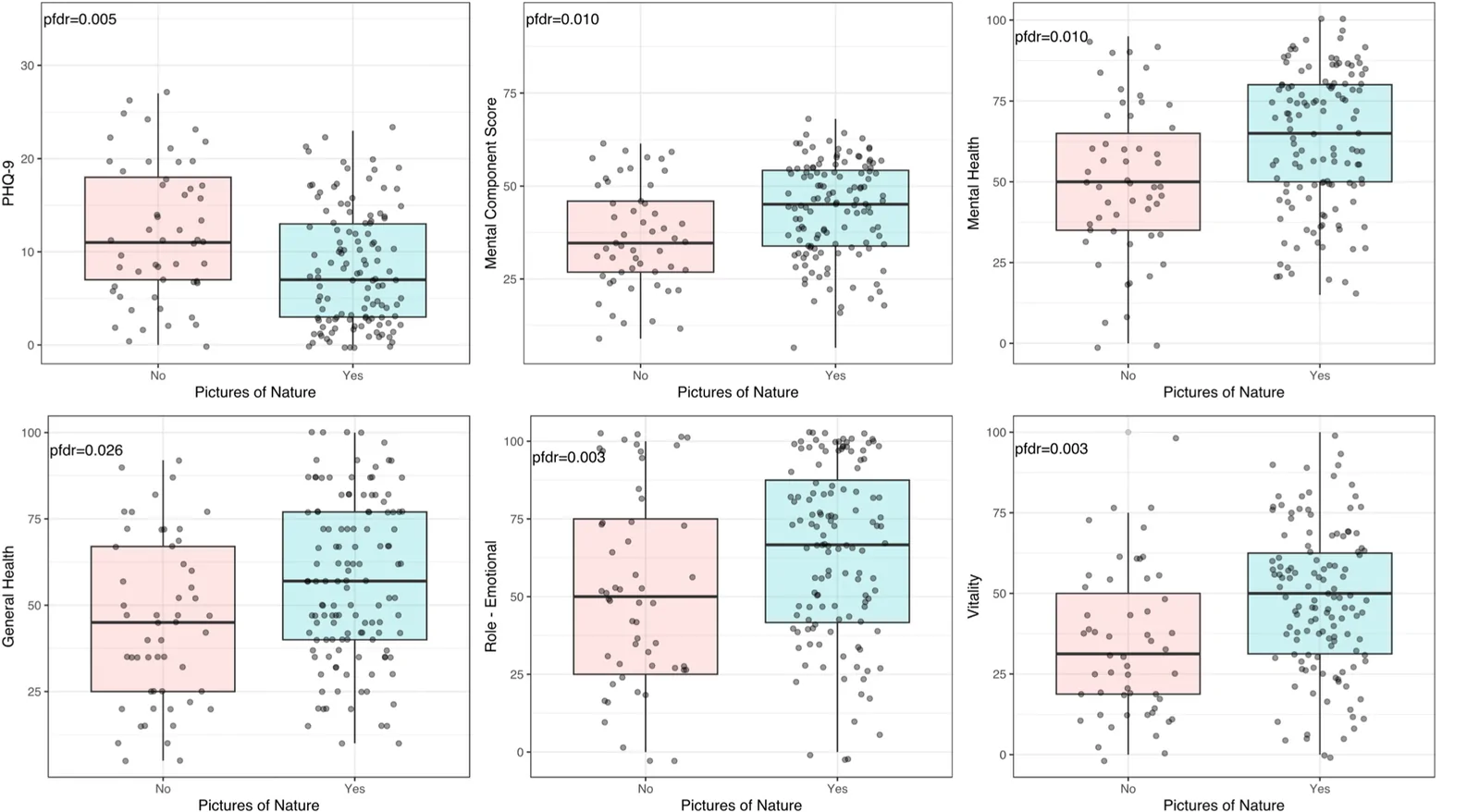
Veteran statistically significant SF-36v2 component scores and PHQ-9 total score to question of having pictures of nature in their homes (Q17). *p*_*fdr*_ determined from pairwise *t*-test, adjusted for multiple testing between the component scores and survey questions through false discovery rates.

**Fig. 6.**
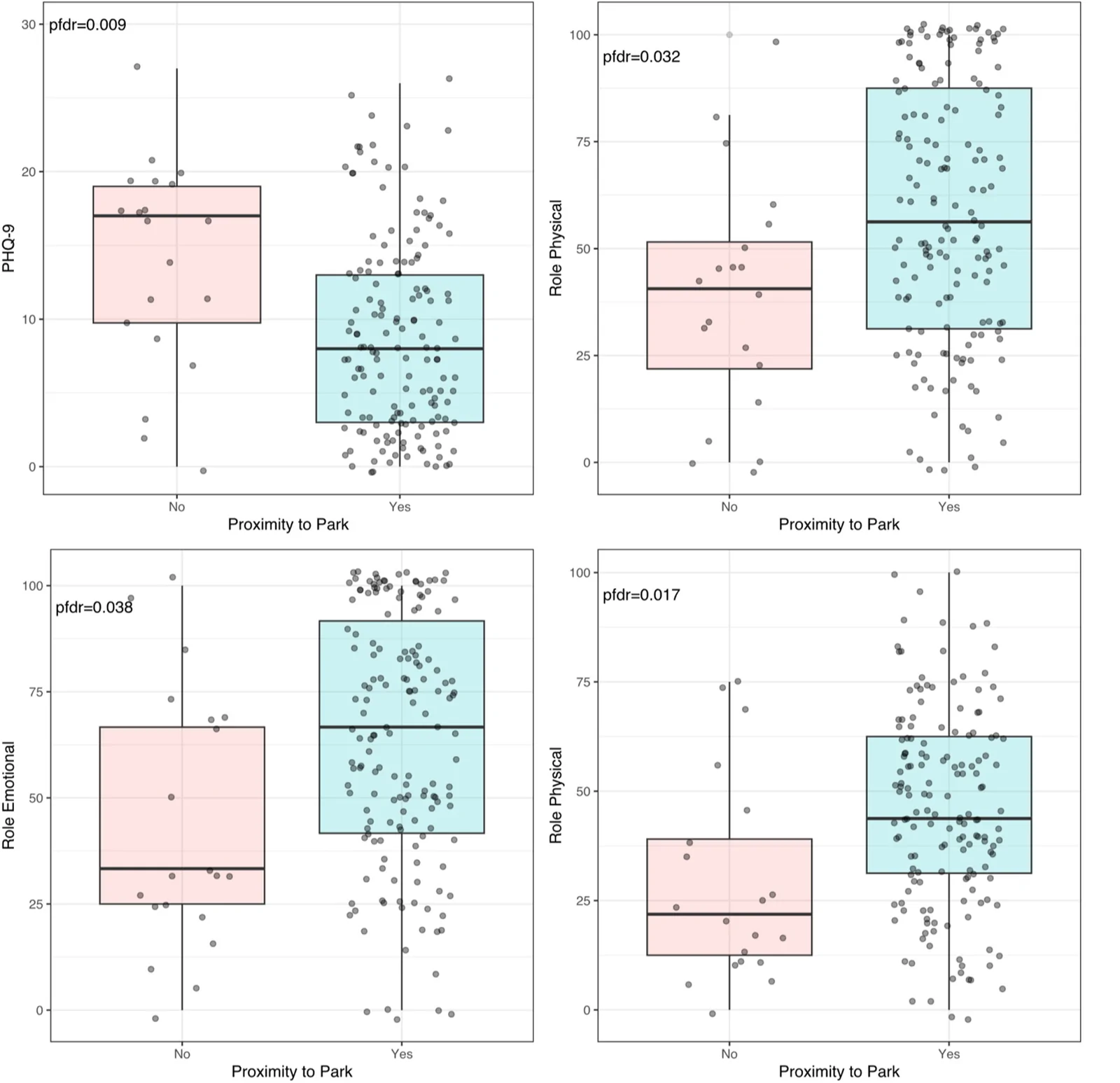
Veteran statistically significant SF-36v2 component scores and PHQ-9 total score to question on living within 0.5 miles to open greenspace (Q13). *p*_*fdr*_ determined from pairwise *t*-test, adjusted for multiple testing between the component scores and survey questions through false discovery rates.

**Table 1:** Theoretically related questions.

Response A	Response B	Theoretical Expectation

Owning a home (Q18)	Major home repair (Q9)	Positive correlation between individuals that own their own home and those that conduct major home repairs
Water damage (Q16)	Have basement (Q15)	Positive correlation between having water damage and residences that have basement
Smoking Indoors (Q6)	Incense (Q7)	Positive correlation between smoking indoors and the use of incense
Square foot residence (Q3)	Type of residence (Q19)	More square foot in single family homes compared to apartments or multiple dwelling units

**Table 2:** Summary of survey results for AF and Veteran Cohorts that participated in the HOME survey.

	All Respondents		Air Force		Veterans	
Questions	Mean	SD	Mean	SD	Mean	SD

How many residences have you lived in over the last 10 years?	4.3	2.9	4.4	2.5	4.2	3.4
How many people currently live in your home with you (including yourself)?	2.4	1.9	2.9	1.5	1.9	2.1
How large in approximate square feet is the home you currently live in?	1828	921	1840	835	1813	1018
What year was your home constructed?	1991	134	1997	98	1983	30
How tall are the ceilings in your home (in feet)?	9.8	2.8	9.9	2.9	9.6	2.5
The overall natural lighting in your home (1–10 scale)	7.2	2.0	7.1	2.0	7.5	2.0
Your ability to adjust your indoor climate to your liking (1–10 scale)	8.1	2.1	8.0	2.2	8.2	2.0
Questions	N	%	N	%	N	%
Do you or anyone smoke any substance in the home you currently live in (e-cigarettes included)?						
Yes	64	15.6 %	13	5.7 %	51	28.3 %
No	346	84.4 %	217	94.3 %	129	71.7 %
Do you burn incense or candles in your home daily?						
Yes	83	20.2 %	43	18.7 %	40	22.2 %
No	327	79.8 %	187	81.3 %	140	77.8 %
Do you have any dogs that are regularly in your home?						
Yes	209	51.1 %	113	49.3 %	96	53.3 %
No	200	48.9 %	116	50.7 %	84	46.7 %
Have you, in the last 5 years, done any of the following in your home: replaced more than 50 % of the carpet, remodeled, replaced the roof?						
Yes	137	33.7 %	76	33.3 %	61	34.1 %
No	270	66.3 %	152	66.7 %	118	65.9 %
Do you have the ability to open the windows in your home whenever you would like?						
Yes	397	97.1 %	221	96.5 %	176	97.8 %
No	12	2.9 %	8	3.5 %	4	2.2 %
Do 50 % or more of the windows in your home look upon a natural setting (e.g. not man-made structures)?						
Yes	187	45.7 %	95	41.5 %	92	51.1 %
No	222	54.3 %	134	58.5 %	88	48.9 %
Do you live within 1 mile of a highway or interstate?						
Yes	220	53.8 %	116	50.7 %	104	57.8 %
No	189	46.2 %	113	49.3 %	76	42.2 %
Do you live within 0.5 miles of open greenspace (park, playground, field, hiking trails, etc)?						
Yes	328	80.2 %	168	73.4 %	160	88.9 %
No	81	19.8 %	61	26.6 %	20	11.1 %
Do you feel you have adequate privacy in your home?						
Yes	369	90.0 %	200	87.0 %	169	93.9 %
No	41	10.0 %	30	13.0 %	11	6.1 %
Does your home have a basement?						
Yes	119	29.0 %	25	10.9 %	94	52.2 %
No	291	71.0 %	205	89.1 %	86	47.8 %
Has your home ever had water damage?						
Yes	106	26.0 %	65	28.6 %	41	22.8 %
No	301	74.0 %	162	71.4 %	139	77.2 %
Do you have any pictures or paintings of nature in your home?						
Yes	272	66.3 %	145	63.0 %	127	70.6 %
No	138	33.7 %	85	37.0 %	53	29.4 %
Do you own or rent the home you live in?						
Own	219	53.7 %	115	50.4 %	104	57.8 %
Rent	189	46.3 %	113	49.6 %	76	42.2 %
What type of home do you live in?						
Detached single family	259	63.3 %	155	67.7 %	104	57.8 %
Multiple dwelling unit (condo, townhouse, etc)	71	17.4 %	20	8.7 %	51	28.3 %
Apartment	61	14.9 %	41	17.9 %	20	11.1 %
Other	18	4.4 %	13	5.7 %	5	2.8 %
If you live in a detached single-family home, what is the size of the lot upon which your home sits?						
less than 0.25 acres	130	46.8 %	94	51.6 %	36	37.5 %
0.26–0.5 acres	81	29.1 %	41	22.5 %	40	41.7 %
0.5–1.0 acres	33	11.9 %	22	12.1 %	11	11.5 %
greater than 1.0 acres	34	12.2 %	25	13.7 %	9	9.4 %

## Appendix A. Supporting information

Supplementary data associated with this article can be found in the online version at doi:10.1016/j.indenv.2025.100103.

## Abbreviations:

AF: Air Force
BASE: Building Assessment Survey and Evaluation
HOME: Housing Occupancy Materials and Environment
CBE: Center for the Built Environment
MCS: Mental Component Score
PCS: Physical Component Score
MIRECC: Rocky Mountain Mental Illness Research Education and Clinical Center
US: United States
US-VMP: United States-Veteran Microbiome Project
