# Supplementary material for: Residential factors associated with mental health in United States Veterans, Air Force military, and Air Force employees
Source: Indoor Environ. Author manuscript; Available in PMC 2026 Oct 17. (PMC13673959; doi:10.1016/j.indenv.2025.100103)
Supplement: Supplementary file 1 [file NIHMS2137162-supplement-Supplemental_File.docx]

**Supplemental Files**

**Supplemental Table 1.** Demographic characteristics for AF and Veteran Cohorts that participated in the HOME survey.

|  | **All Respondents** | | **Air Force** | | **Veterans** | |  |
| --- | --- | --- | --- | --- | --- | --- | --- |
|  | **N** | **%** | **N** | **%** | **N** | **%** | |
| Variable | 410 |  | 230 |  | 180 |  | |
| Age Category |  |  |  |  |  |  | |
| 20-29 | 72 | 17.6% | 57 | 24.8% | 15 | 8.3% | |
| 30-39 | 124 | 30.2% | 80 | 34.8% | 44 | 24.4% | |
| 40-49 | 75 | 18.3% | 38 | 16.5% | 37 | 20.6% | |
| 50-59 | 79 | 19.3% | 42 | 18.3% | 37 | 20.6% | |
| 60-69 | 46 | 11.2% | 13 | 5.7% | 33 | 18.3% | |
| 70+ | 14 | 3.4% | 0 | 0.0% | 14 | 7.8% | |
| Sex |  |  |  |  |  |  | |
| Male | 315 | 77.2% | 175 | 76.4% | 140 | 78.2% | |
| Female | 93 | 22.8% | 54 | 23.6% | 39 | 21.8% | |
| Race |  |  |  |  |  |  | |
| Caucasian | 314 | 77.0% | 180 | 78.3% | 134 | 75.3% | |
| African American | 34 | 8.3% | 16 | 7.0% | 18 | 10.1% | |
| Multiracial | 23 | 5.6% | 10 | 4.3% | 13 | 7.3% | |
| Other | 37 | 9.1% | 24 | 10.4% | 13 | 7.3% | |
| Ethnicity |  |  |  |  |  |  | |
| Hispanic | 47 | 11.8% | 24 | 10.9% | 23 | 13.0% | |
| Non-Hispanic | 351 | 88.2% | 197 | 89.1% | 154 | 87.0% | |
| Marital Status |  |  |  |  |  |  | |
| Married | 253 | 61.7% | 166 | 72.2% | 87 | 48.3% | |
| Single | 75 | 18.3% | 40 | 17.4% | 35 | 19.4% | |
| Cohabitating | 20 | 4.9% | 7 | 3.0% | 13 | 7.2% | |
| Separated/Divorced | 56 | 13.7% | 16 | 7.0% | 40 | 22.2% | |
| Widowed | 6 | 1.5% | 1 | 0.4% | 5 | 2.8% | |
| Education |  |  |  |  |  |  | |
| Some high school, no diploma | 1 | 0.2% | 1 | 0.4% | 0 | 0.0% | |
| High school diploma or equivalent (GED) | 23 | 5.6% | 7 | 3.0% | 16 | 8.9% | |
| Some college credit, no degree | 102 | 24.9% | 47 | 20.4% | 55 | 30.6% | |
| Associate degree | 87 | 21.2% | 55 | 23.9% | 32 | 17.8% | |
| Bachelor's degree | 109 | 26.6% | 64 | 27.8% | 45 | 25.0% | |
| Master's degree | 82 | 20.0% | 54 | 23.5% | 28 | 15.6% | |
| Doctorate Degree | 6 | 1.5% | 2 | 0.9% | 4 | 2.2% | |
| Employment |  |  |  |  |  |  | |
| Employed full time | 275 | 67.7% | 228 | 99.6% | 47 | 26.6% | |
| Employed part time | 21 | 5.2% | 1 | 0.4% | 20 | 11.3% | |
| Unemployed, seeking employment | 38 | 9.4% | 0 | 0.0% | 38 | 21.5% | |
| Unemployed, not currently seeking employment | 17 | 4.2% | 0 | 0.0% | 17 | 9.6% | |
| Retired from the workforce | 55 | 13.5% | 0 | 0.0% | 55 | 31.1% | |
| School Enrollment |  |  |  |  |  |  | |
| Not in School | 326 | 79.9% | 175 | 76.4% | 151 | 84.4% | |
| Part time student | 55 | 13.5% | 47 | 20.5% | 8 | 4.5% | |
| Full time student | 27 | 6.6% | 7 | 3.1% | 20 | 11.2% | |
| Ever Homeless |  |  |  |  |  |  | |
| Yes | 46 | 12.0% | 8 | 3.5% | 38 | 24.8% | |
| No | 337 | 88.0% | 222 | 96.5% | 115 | 75.2% | |

**Supplemental Table 2.** Air Force Correlation Matrix

**Supplemental Table 3.** Veteran Correlation Matrix

**Supplemental Figure 1.** Principal component analysis (PCA) for combination of AF and Veteran cohorts with (A) percent of questions in each dimension, (B) quality of representation across the two most prevalent dimensions, and (C) PCA figure of the two most prevalent dimensions.

The HOME survey (V2) is included in this supplemental. This is identical to the survey that was taken by participants in this study with exception of question 4 that was open answer choice in V1.

Questions were selected based on previous research on buildings and mental health plus researcher perceived factors of importance. The following outlines a rationale for questions with how they might be associated with mental health outcomes, based primarily on our previous works.^1-6^

**Supplemental Table 4.** HOME Questions and Rationale for Inclusion

| **Question** | **Connection to Mental Health Outcomes** |
| --- | --- |
| 1. How many residences have you lived in over the last 10 years? | Housing instability risk factor for depression^7^. |
| 2. How many people currently live in the home with you? | Increased density of individuals in home risk factor for depression^7,8^. |
| 3. How large in approximate square feet is the home you live in? | Potential connection to social economic status that is well documented as a risk factor for negative mental health^9,10^. Smaller homes have higher air exchange rates^11^ (see discussion on air exchange rate and mental health in Hoisington et al., 2019) |
| 4. What year was your home constructed? | Older homes are more likely to have mold issues^12^ and have higher air exchange rates^11^ (see discussion on air exchange rate and mental health in Hoisington et al., 2019) |
| 5. How tall are the ceilings in your home? | Potential connection to social economic status, well documents a risk factor for negative mental health^9,10^ |
| 6. Do you or anyone smoke any substance in the home you live in (e-cigarettes included)? | Independently, smoking can be a risk factor for range of psychiatric conditions^13,14^ and smoking causes increase in indoor pollutants^15^ (see summary of criteria pollutant exposure and mental health in Hoisington et al, 2024) |
| 7. Do you burn incense or candles in your home daily? | Increase in volatile organic compounds and particulates^16^, particularly fine particulate matter (under 2.5 μm)^17,18^ that can lead to worse mental health outcomes (see summary of criteria pollutant exposure and mental health in Hoisington et al, 2024) |
| 8. Do you have any dogs that are regularly in your home? | Direct relationship is complex.^19^ Indirectly, mental health outcomes that could improve related to dog ownership are increased physical activity^20^, more social interactions^21^, and additional time in nature (see Questions 13 and 17). |
| 9. Have you in the last 5 years done any of the following: replaced more than 50% of the carpet, applied paint to more than 50% of your home, remodeled, or replaced the roofing of your home? | Poor housing conditions associated with worse mental health^22,23^ |
| 10. Do you have the ability to open the windows in your home whenever you would like? | Ability to have person control of their surroundings results in improved mental health^24,25^. Windows also for viewing nature (see Questions 13 and 17) and lighting (see Question 20) |
| 11. Do 50% or more of the windows in your home look upon a natural setting (e.g. not man-made structures)? | Viewing nature associated with less frequent use of health care services^26^, reduced nervousness and anxiety^27^, lower depression^28-30^, and reduced stress^27,31,32^ |
| 12. Do you live within 1 mile of a highway or interstate? | Effects on mental health could be from increased noise from roadways^4^ (141) that is an annoyance^33-35^ or increased levels of airborne pollutants (see summary of criteria pollutant exposure and mental health in Hoisington et al, 2024) |
| 13. Do you live within 0.5 miles of open greenspace (park, playground, field, hiking trails, etc.)? | Greenspace around homes associated lower symptoms for depression, anxiety, and stress^36^. Although well studied (see systematic review that highlights heterogeneity among studies^37^), factors likely influencing results to include age and sex.^38^ |
| 14. Do you feel you have adequate privacy in your home? | Inadequate privacy associated with increased stress (see review by Ferreira de Macedo et al., 2022) |
| 15. Does your home have a basement? | Basements can be area with low ventilation, high dampness, and less occupance that leads to mold growth^40,41^ (see Question 16 for connection of mold and mental health) |
| 16. Has your home ever had water damage? | Water damage can lead to mold. Mold can have dose-response to mental health outcomes (e.g. increased mold had higher depression rates)^42^ |
| 17. Do you have any pictures or paintings of nature in your home? | Pictures of nature could have restorative effect^43-45^ or could reduce stress through psychophysiological pathways^31,46,47^ |
| 18. Do you own or rent the home you live in? | Home stability from owning versus renting can have improved mental health^40^ |
| 19. What type of home do you live in? | Detached homes have higher odds of improved mental health outcomes for occupants^48^ |
| Detached Home: what size is  the lot upon which your home  sit? | Larger lots may be associated with increased greenspace and connection to nature (positive, see Question 13) but could also result in more social isolation |
| Apartment: what floor do you  live on? | Higher floors negatively associated with mental health symptoms^49^ that might be caused from lack of social interactions^50^ |
| 20. The overall natural lighting in your home: | Building occupants prefer natural over artificial light^51,52^ and inadequate daylights increases risk of depression^53^ |
| 21. Your ability to adjust your indoor climate to your liking: | Thermal discomfort associated with increased stress^54^ and psychological restoration^55^ |

**References**

1	Brenner, L. A. *et al.* Military-Related Exposures, Social Determinants of Health, and Dysbiosis: The United States-Veteran Microbiome Project (US-VMP). *Front Cell Infect Microbiol* **8**, 400, doi:10.3389/fcimb.2018.00400 (2018).

2	Hoisington, A. J., Brenner, L. A., Kinney, K., A., Postolache, T., T. & Lowry, C. A. The microbiome of the built environment and mental health. *Microbiome* **3**, 60, doi:10.1186/s40168-015-0127-0 (2015).

3	Hoisington, A. J., Stearns-Yoder, K. A., Kovacs, E. J., Postolache, T. T. & Brenner, L. A. Airborne Exposure to Pollutants and Mental Health: A Review with Implications for United States Veterans. *Current Environmental Health Reports* **Accepted for Publication** (2024).

4	Hoisington, A. J. *et al.* Ten questions concerning the built environment and mental health. *Building and Environment* **155**, 58-69, doi:<https://doi.org/10.1016/j.buildenv.2019.03.036> (2019).

5	Taylor, W. L. *et al.* A framework for estimating the United States depression burden attributable to indoor fine particulate matter exposure. *Science of The Total Environment* **756**, 143858, doi:<https://doi.org/10.1016/j.scitotenv.2020.143858> (2021).

6	Beemer, C. J. *et al.* A brief review on the mental health for select elements of the built environment. *Indoor and Built Environment* **30**, 152 - 165 (2019).

7	Suglia, S. F., Duarte, C. S. & Sandel, M. T. Housing Quality, Housing Instability, and Maternal Mental Health. *Journal of Urban Health* **88**, 1105-1116, doi:10.1007/s11524-011-9587-0 (2011).

8	Ruiz-Tagle, J. & Urria, I. Household overcrowding trajectories and mental well-being. *Social Science & Medicine* **296**, 114051, doi:<https://doi.org/10.1016/j.socscimed.2021.114051> (2022).

9	Feinstein, J. S. The relationship between socioeconomic status and health: a review of the literature. *The Milbank quarterly* **71**, 279-322 (1993).

10	Reiss, F. *et al.* Socioeconomic status, stressful life situations and mental health problems in children and adolescents: Results of the German BELLA cohort-study. *PLoS One* **14**, e0213700, doi:10.1371/journal.pone.0213700 (2019).

11	Chan, W. R., Nazaroff, W. W., Price, P. N., Sohn, M. D. & Gadgil, A. J. Analyzing a database of residential air leakage in the United States. *Atmospheric Environment* **39**, 3445-3455, doi:<https://doi.org/10.1016/j.atmosenv.2005.01.062> (2005).

12	Reponen, T. *et al.* Family and home characteristics correlate with mold in homes. *Environ Res* **124**, 67-70, doi:10.1016/j.envres.2013.04.003 (2013).

13	Taylor, G. M. J. & Munafò, M. R. Does smoking cause poor mental health? *The Lancet Psychiatry* **6**, 2-3, doi:10.1016/S2215-0366(18)30459-0 (2019).

14	Fluharty, M., Taylor, A. E., Grabski, M. & Munafò, M. R. The Association of Cigarette Smoking With Depression and Anxiety: A Systematic Review. *Nicotine Tob Res* **19**, 3-13, doi:10.1093/ntr/ntw140 (2017).

15	Jones, A. P. Indoor air quality and health. *Atmospheric Environment* **33**, 4535-4564, doi:10.1016/s1352-2310(99)00272-1 (1999).

16	Lung, S.-C. C. & Hu, S.-C. Generation rates and emission factors of particulate matter and particle-bound polycyclic aromatic hydrocarbons of incense sticks. *Chemosphere* **50**, 673-679 (2003).

17	Jetter, J. J., Guo, Z., McBrian, J. A. & Flynn, M. R. Characterization of emissions from burning incense. *Science of the Total Environment* **295**, 51-67 (2002).

18	Hien, T. T. *et al.* Characterization of Particulate Matter (PM1 and PM2.5) from Incense Burning Activities in Temples in Vietnam and Taiwan. *Aerosol and Air Quality Research* **22**, 220193, doi:10.4209/aaqr.220193 (2022).

19	Liu, S. *et al.* Is Dog Ownership Associated with Mental Health? A Population Study of 68,362 Adults Living in England. *Anthrozoös* **32**, 729-739, doi:10.1080/08927936.2019.1673033 (2019).

20	Christian, H. E. *et al.* Dog ownership and physical activity: a review of the evidence. *J Phys Act Health* **10**, 750-759 (2013).

21	Wood, L. *et al.* The Pet Factor - Companion Animals as a Conduit for Getting to Know People, Friendship Formation and Social Support. *PLOS ONE* **10**, e0122085, doi:10.1371/journal.pone.0122085 (2015).

22	Pevalin, D. J., Reeves, A., Baker, E. & Bentley, R. The impact of persistent poor housing conditions on mental health: A longitudinal population-based study. *Preventive medicine* **105**, 304-310 (2017).

23	Rollings, K. A., Wells, N. M., Evans, G. W., Bednarz, A. & Yang, Y. Housing and neighborhood physical quality: Children's mental health and motivation. *Journal of Environmental Psychology* **50**, 17-23, doi:<https://doi.org/10.1016/j.jenvp.2017.01.004> (2017).

24	Miller, S. M. Controllability and human stress: Method, evidence and theory. *Behaviour research and therapy* **17**, 287-304 (1979).

25	Barnes, R. D. Perceived freedom and control in the built environment. *Cognition, social behavior, and the environment* (1981).

26	Edwards, L. & Torcellini, P. Literature Review of the Effects of Natural Light on Building Occupants. Report No. NREL/TP-550-30769; TRN: US200401%%275 United States 10.2172/15000841 TRN: US200401%%275 NREL English, Medium: ED; Size: 58 pages (; National Renewable Energy Lab., Golden, CO. (US), 2002).

27	Rosenthal, N. E. *et al.* Seasonal affective disorder: a description of the syndrome and preliminary findings with light therapy. *Archives of general psychiatry* **41**, 72-80 (1984).

28	Ulrich, R. S. Natural Versus Urban Scenes:Some Psychophysiological Effects. *Environment and Behavior* **13**, 523-556, doi:10.1177/0013916581135001 (1981).

29	Berto, R. Exposure to restorative environments helps restore attentional capacity. *Journal of Environmental Psychology* **25**, 249-259, doi:10.1016/j.jenvp.2005.07.001 (2005).

30	Kaplan, R. & Kaplan, S. *The experience of nature: A psychological perspective*. (CUP Archive, 1989).

31	Randall, K., Shoemaker, C. & Relf, D. G. in *The Role of Horticulture in Human Well-Being and Social Development: A National Symposium, Arlington, Virginia. Timber Press, Portland, Oregon.* 106-109.

32	Wells, N. M. & Evans, G. W. Nearby Nature:A Buffer of Life Stress among Rural Children. *Environment and Behavior* **35**, 311-330, doi:10.1177/0013916503035003001 (2003).

33	Hammersen, F., Niemann, H. & Hoebel, J. Environmental Noise Annoyance and Mental Health in Adults: Findings from the Cross-Sectional German Health Update (GEDA) Study 2012. *International Journal of Environmental Research and Public Health* **13**, 954 (2016).

34	Beutel, M. E. *et al.* Noise annoyance is associated with depression and anxiety in the general population-the contribution of aircraft noise. *Plos one* **11**, e0155357 (2016).

35	Jensen, H. A., Rasmussen, B. & Ekholm, O. Neighbour and traffic noise annoyance: a nationwide study of associated mental health and perceived stress. *European journal of public health* (2018).

36	Beyer, K. *et al.* Exposure to Neighborhood Green Space and Mental Health: Evidence from the Survey of the Health of Wisconsin. *International Journal of Environmental Research and Public Health* **11**, 3453 (2014).

37	Gascon, M. *et al.* Mental health benefits of long-term exposure to residential green and blue spaces: a systematic review. *International journal of environmental research and public health* **12**, 4354-4379 (2015).

38	Thomas, A.-B., Richard, M. & Terry, H. The association between green space and mental health varies across the lifecourse. A longitudinal study. *Journal of Epidemiology and Community Health* **68**, 578, doi:10.1136/jech-2013-203767 (2014).

39	de Macedo, P. F., Ornstein, S. W. & Elali, G. A. Privacy and housing: research perspectives based on a systematic literature review. *J Hous Built Environ* **37**, 653-683, doi:10.1007/s10901-022-09939-z (2022).

40	Wood, G. A., Clark, W. A. V., Ong ViforJ, R., Smith, S. J. & Truong, N. T. K. Residential mobility and mental health. *SSM - population health* **21**, 101321, doi:<https://doi.org/10.1016/j.ssmph.2022.101321> (2023).

41	Afanou, A. K., Straumfors, A. & Eduard, W. Fungal aerosol composition in moldy basements. *Indoor air* **29**, 780-790 (2019).

42	Shenassa, E. D., Daskalakis, C., Liebhaber, A., Braubach, M. & Brown, M. Dampness and mold in the home and depression: an examination of mold-related illness and perceived control of one's home as possible depression pathways. *Am J Public Health* **97**, 1893-1899, doi:10.2105/ajph.2006.093773 (2007).

43	Ulrich, R. S. Biophilia, biophobia, and natural landscapes. *The biophilia hypothesis* **7**, 73-137 (1993).

44	Kaplan, S. The restorative benefits of nature: Toward an integrative framework. *Journal of Environmental Psychology* **15**, 169-182, doi:<https://doi.org/10.1016/0272-4944(95)90001-2> (1995).

45	Kaplan, R. The Nature of the View from Home. *Environment and Behavior* **33**, 507-542, doi:10.1177/00139160121973115 (2016).

46	Chang, C.-Y. & Chen, P.-K. Human Response to Window Views and Indoor Plants in the Workplace. *HortScience* **40**, 1354-1359 (2005).

47	Ulrich, R. S. *et al.* Stress recovery during exposure to natural and urban environments. *Journal of environmental psychology* **11**, 201-230 (1991).

48	Ghimire, J., Carswell, A. T., Ghimire, R. & Turner, P. R. The Impact of U.S. Housing Type and Residential Living Situations on Mental Health during COVID-19. *International Journal of Environmental Research and Public Health* **18**, 8281 (2021).

49	Hannay, D. Mental health and high flats. *Journal of chronic diseases* **34**, 431-432 (1981).

50	Fanning, D. Families in flats. *British Medical Journal* **4**, 382 (1967).

51	Veitch, J. A., Hine, D. W. & Gifford, R. END USERS‘ KNOWLEDGE, BELIEFS, and PREFERENCES FOR LIGHTING. *Journal of Interior Design* **19**, 15-26, doi:doi:10.1111/j.1939-1668.1993.tb00159.x (1993).

52	Heerwagen, J. H. H., R. Lighting and Psychological Comfort. *Lighting Design and Application* **6**, 47* (1986).

53	Organization, W. H. Large analysis and review of European housing and health status (LARES). (World Health Organization, Copenhagen, Denmark, 2007).

54	Engineer, A. *et al.* An integrative health framework for wellbeing in the built environment. *Building and Environment* **205**, 108253 (2021).

55	Bai, Y. & Jin, H. Effects of visual, thermal, and acoustic comfort on the psychological restoration of the older people in a severe cold city. *Building and Environment* **239**, 110402, doi:<https://doi.org/10.1016/j.buildenv.2023.110402> (2023).
